# Risk factors for early morbidity and mortality following pancreatoduodenectomy with concomitant vascular reconstruction

**DOI:** 10.1016/j.amsu.2021.102587

**Published:** 2021-07-27

**Authors:** Tiffany Y. Lim, I. Michael Leitman

**Affiliations:** Department of Surgery, Icahn School of Medicine at Mount Sinai, New York, NY, United States

**Keywords:** Pancreatic resection, Portal vein, Superior mesenteric artery, Pancreas, Carcinoma

## Abstract

**Background:**

Locally advanced pancreatic tumors may require vascular reconstruction for complete resection. However, pancreatoduodenectomy with vascular resection (PDVR) remains a subject of debate due to increased complications.

**Methods:**

Patients were identified using the ACS NSQIP Participant User Data Files from 2014 to 2019.

**Results:**

The 30-day mortality rate was 2.7%; major complications occurred in 32.2%. There is an increasing trend of PDVR in patients requiring pancreatectomy. There were no significant differences in mortality between PDVR with vein, artery, or venous and arterial resections. High BMI and postoperative biliary stent were risk factors for early complications. High BMI and COPD increased risk of early mortality. Chemotherapy and chemoradiotherapy were negative predictors for early morbidities and mortality, respectively.

**Conclusion:**

This study identifies the predictors of early morbidity and mortality in PDVR. The results of this study may assist decision making in perioperative management to optimize overall survival and guide additional research.

## Highlights

• Pancreaticoduodenectomy with concomitant vascular reconstruction (PDVR) for carcinoma is increasing in frequency for locally advanced pancreatic neoplasms.
• Early morbidity and mortality rates are acceptable.
• Patients with elevated body mass index (BMI) and underlying COPD have a higher mortality.
• Elevated BMI and preoperative biliary stenting are associated with a higher incidence of early complications.
• Neoadjuvant chemotherapy and chemoradiation may also improve resectability and post-operative outcomes.

## 1 Introduction

Pancreatic cancer has the lowest 5-year survival rate (10%) among the most common types of cancer and its mortality rate continued to increase by 0.3% since 2000 [1]. While complete resection with negative margin has shown the most significant benefit in long-term survival, not all pancreatic tumors are resectable [[2], [3], [4], [5]]. In the most recent National Comprehensive Cancer Network (NCCN) Guidelines (version 1.2021), a resectable tumor was defined as “no arterial tumor contact (celiac axis, superior mesenteric artery, or common hepatic artery)” and/or “no tumor contact with the superior mesenteric vein or portal vein or ≤ 180° contact without vein contour irregularity.” [6] However, due to the lack of effective screening program and natural history of the disease; patients commonly remain asymptomatic until they present with a locally advanced tumor [2].

Historically, involvement of adjacent vasculature was considered a contraindication for resection. Earlier studies of neoadjuvant therapy failed to show sufficient survival benefit and tumor shrinkage and even increased rates of complications [7]. However, more recent neoadjuvant therapy trials have shown survival benefits and increased in R0 resection rate [[8], [9], [10], [11]]. As a result of the success with neoadjuvant therapy regimens and operative technical improvement, pancreatoduodenectomy with vascular resection (PDVR) has been increasingly utilized and recommended for patients with borderline resectable pancreatic cancer [6,12].

Despite the increase in utilization, there are conflicting results regarding the safety and efficacy of PDVR. One meta-analysis study that consolidated several studies with smaller sample sizes showed that PDVR, specifically superior mesenteric arterial (SMA) resection, results in a higher mortality rate at 1-year and 3-years compared to pancreatoduodenectomy without SMA resection [13]. Worni analyzed a large multi-institutional database, Nationwide Inpatient Sample, from 2000 to 2009, and showed no significant difference for in-hospital mortality with PDVR compared to pancreatoduodenectomy alone. Interestingly, in the same study, PDVR had significantly higher in-hospital mortality in the highest hospital volume quartile [14]. In contrast, another study utilizing The American College of Surgeons National Surgical Quality Improvement Program (ACS-NSQIP) database showed a significant increase in 30-day mortality following PDVR [15]. A smaller study investigated long-term outcome and found no significant survival differences between PDVR and standard PD at one year and three years post-op but a significantly lower survival rate in PDVR at five years post-op. Regardless, the survival rate after PDVR was significantly higher than patients who received palliative chemoradiation without surgery [16].

To date, there are no published studies utilizing a multi-institutional database to investigate specific perioperative patient-specific and modifiable risk factors that predispose to early complications and mortality in PDVR. In addition, previous studies have been limited in comparing outcomes in different types of vascular resection in PDVR. Therefore, the aims of this study were to (1) assess trend of utilization of PDVR and its complications using a multi-institutional, risk-adjusted database from 2014 to 2019, and (2) identify modifiable perioperative factors that predispose to greater risks for early morbidity and mortality after PDVR.

## 2 Methods

### 2.1 Data Collection and patient selection

The American College of Surgeons National Surgical Quality Improvement Program (ACS-NSQIP) Participant User Files and Procedure Targeted Files from 2014 to 2019 were analyzed retrospectively. The ACS-NSQIP provided an extensive pre-operative and peri-operative dataset as well as risk-adjusted 30-day postoperative morbidity and mortality. All data were recorded by trained personnel from over 700 healthcare institutions. There were no patient or hospital identifiers included in the Participant User Files. This project received human research exempt determination by the institutional review board (IRB-20-02957).

Patients who underwent pancreatoduodenectomy were identified using the Current Procedural Terminology (CPT) codes 48150, 48152, 48153, 48154, 48155. Patients who were identified to have disseminated cancer preoperatively were excluded as they are typically identified as non-surgical candidates. Patients with postoperative diagnosis specific to pancreatic cancer were selected using the International Classification of Diseases (ICD-9 and ICD-10), including benign neoplasm of pancreas (D13.6), benign neoplasm of pancreas except islets of Langerhans (211.6), malignant neoplasm of pancreas (C25), malignant neoplasm of head of pancreas (157, C25.0), malignant neoplasm of body of pancreas (157.1, C25.1), malignant neoplasm of tail of pancreas (157.2, C25.2), malignant neoplasm of pancreatic duct (157.3, C25.3), malignant neoplasm of endocrine pancreas (157.4, C25.4), Malignant neoplasm of other specified sites of pancreas (157.8, C25.7), malignant neoplasm of unspecified pancreas (157.9, C25.9), malignant neoplasm of overlapping sites of pancreas (C25.8). Information of types of vascular resections were extracted from procedure targeted files. All dataset preparation and statistical analyses were performed using R studio Desktop (Version 1.1.463 - Vienna, Austria) [17]. This work has been reported in line with the STROCSS criteria [18].

The American College of Surgeons National Surgical Quality Improvement Program and the hospitals participating in the ACS NSQIP are the source of the data used herein; they have not verified and are not responsible for the statistical validity of the data analysis or the conclusions derived by the authors.

### 2.2 Outcomes of interest

30-day mortality and major morbidities were the primary outcomes of interest. Major morbidities included occurrences of any of the following: dehiscence, stroke, cardiac arrest requiring CPR, myocardial infarction, pneumonia, dependence on a mechanical ventilator for more than 48 h, unplanned reintubation, acute renal failure, progressive renal insufficiency, sepsis, septic shock, superficial incisional surgical site infection, deep incisional surgical site infection, organ space surgical site infection, pulmonary embolism, deep vein thrombosis (DVT) and return to operation room.

### 2.3 Statistical analysis

Chi-square and ANOVA analyses were used to investigate significance of patient demographic and comorbid conditions between three postoperative outcome groups – uncomplicated, early morbidities but survival at 30 days, and 30-day mortality. Post-hoc univariate analyses were performed to identify specific outcome groups that were significant.

Multivariable logistic regression models adjusted for types of vascular resections and comorbidities were constructed to identify perioperative risk factors that significantly increase the probability of developing mortality and major morbidities after PDVR. Modifiable risk factors with adequate sample size were selected when building multivariable logistic regression models.

All tests of significance were determined at *p*-value < 0.05.

## 3 Results

**Fig. 1:**
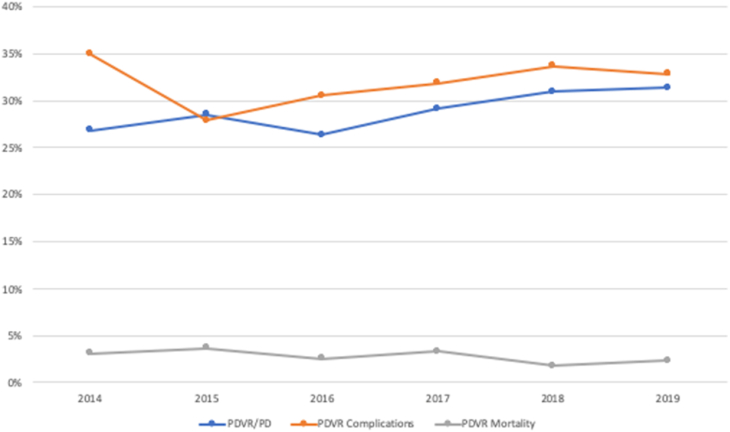
Trend of PDVR Utilization, Major Complications and Mortality. Chi-square analysis showed significant increase in percentages of PDVR among all pancreatoduodenectomy cases from 2014 to 2019 in the NSQIP database (P = 0.022). No significant changes were observed in postoperative mortality (p = 0.150) or morbidities (p = 0.417).

From 2014 to 2019, there were 13,479 patients in NSQIP database that underwent pancreatoduodenectomy for pancreatic cancer, of which 3146 underwent PDVR. The percentage of PDVR among patients who underwent resection for pancreatic cancer significantly increased from 21.7% in 2014 to 24.8% in 2019 (p = 0.022). The 30-day mortality rate was 2.7%. Major complications without mortality occurred in 32.2%. There were no significant trends observed early mortality (p = 0.150) or early morbidities (p = 0.417) during the study period (Fig. 1).

A comparison of the demographic and comorbid characteristics between patients and postoperative outcomes in provided in Table 1. There were significant differences among the three postoperative outcome groups (no complications, early morbidity, and postoperative mortality) in patients for BMI, proportion of COPD, use of anti-hypertension medication, and preoperative biliary stent placement. Post-hoc analyses reveals that patient with an uncomplicated postoperative course had a higher proportion of preoperative chemotherapy. There was also a significant increase in proportion of patients that developed early morbidity in patients that underwent neoadjuvant chemoradiotherapy.

**Table 1:** Demographics and patient characteristics of patients underwent PDVR.

Patient Characteristics		Total (N = 3146)		No complications (n = 2050)		Morbidity (n = 1012)		Death (n = 84)		p value
		n	%	n	%	n	%	n	%	
Age		65.95		66.04		65.59		67.98		0.093
BMI		26.46		26.17		26.92		28.24		<0.001
Race										0.135
	White	2226	70.76%	1474	71.90%	685	67.69%	67	79.76%	
	Black or African American	214	6.80%	138	6.73%	73	7.21%	3	3.57%	
	American Native or Alaska Native	5	0.16%	4	0.20%	1	0.10%	0	0.00%	
	Asian	150	4.77%	102	4.98%	45	4.45%	3	3.57%	
	Native Hawaiian or Pacific Islander	5	0.16%	2	0.10%	3	0.30%	0	0.00%	
	Others/Unidentified	546	17.36%	330	16.10%	205	20.26%	11	13.10%	
Female		1545	49.11%	1038	50.63%	462	45.65%	45	53.57%	0.025
Smoke		547	17.39%	350	17.07%	178	17.59%	19	22.62%	0.413
Diabetes		1014	32.23%	646	31.51%	339	33.50%	29	34.52%	0.489
Ascites		14	0.45%	8	0.39%	6	0.59%	0	0.00%	0.602
HTN medication		1655	52.61%	1040	50.73%	558	55.14%	57	67.86%	0.001
COPD		127	4.04%	70	3.41%	47	4.64%	10	11.90%	<0.001
Transfusion		28	0.89%	18	0.88%	8	0.79%	2	2.38%	0.327
Bleeding Disorder		115	3.66%	69	3.37%	43	4.25%	3	3.57%	0.472
Jaundice		1412	44.88%	906	44.78%	460	46.00%	46	54.76%	0.181
Preoperative biliary stent		1947	61.89%	1226	61.98%	665	67.10%	56	72.73%	0.006
Neoadjuvant therapy										<0.001
	Chemotherapy	881	28.00%	615	30.00%	244	24.11%	22	26.19%	
	Radiotherapy	74	2.35%	43	2.10%	28	2.77%	3	3.57%	
	Chemoradiotherapy	612	19.45%	377	18.39%	227	22.43%	8	9.52%	
	None	1579	50.19%	1015	49.51%	513	50.69%	51	60.71%	

Intraoperative findings were compared to examine the impact on postoperative outcomes (Table 2). There were no significant differences between postoperative outcomes in terms of TMN stage or duct size. Post-hoc analyses revealed that there were significantly less patients who had an uncomplicated postoperative course among patients with a “soft” gland texture.

**Table 2:** Intraoperative characteristics.

		No Complications		Morbidity		Death		p value
		n	%	n	%	n	%	
T stage								0.414
	T0	13	0.66%	11	1.13%	0	0.00%	
	T1	202	10.22%	93	9.58%	5	6.67%	
	T2	539	27.26%	276	28.42%	16	21.33%	
	T3	1175	59.43%	559	57.57%	51	68.00%	
	T4	44	2.23%	27	2.78%	3	4.00%	
	Tis	4	0.20%	5	0.51%	0	0.00%	
M Stage								0.708
	M0	1443	97.90%	716	97.55%	55	96.49%	
	M1	31	2.10%	18	2.45%	2	3.51%	
N Stage								0.411
	N0	678	34.52%	346	35.67%	30	40.00%	
	N1	1179	60.03%	568	58.56%	38	50.67%	
	N2	107	5.45%	56	5.77%	7	9.33%	
Gland Texture								0.042
	Soft	298	20.03%	165	25.11%	16	31.37%	
	Intermediate	205	13.78%	89	13.55%	6	11.76%	
	Hard	985	66.20%	403	61.34%	29	56.86%	
Duct Size								0.060
	<3 mm	284	18.39%	164	23.60%	10	19.61%	
	3–6 mm	918	59.46%	398	57.27%	31	60.78%	
	>6 mm	342	22.15%	133	19.14%	10	19.61%	

Rates for complications were compared between different types of vascular resection – vein, artery, and vein and artery (Table 3). There were significant differences of occurrences of myocardial infarction and DVT amongst different types of vascular resection.

**Table 3:** Early complications in PDVR with vein, artery and vein and artery resection.

Complication	Total		Vein		Artery		Vein and artery		p-value
	n	%	n	%	n	%	n	%	
Death	84	2.7%	55	2.29%	11	4.37%	18	3.65%	0.051
Stroke	16	0.5%	11	0.46%	3	1.19%	2	0.41%	0.281
Myocardial Infarction	40	1.3%	37	1.54%	3	1.19%	0	0.00%	0.021
Pneumonia	132	4.2%	94	3.92%	16	6.35%	22	4.46%	0.177
On Ventilator greater than 48 Hours	131	4.2%	100	4.16%	15	5.95%	16	3.25%	0.216
Unplanned reintubation	112	3.6%	78	3.25%	15	5.95%	19	3.85%	0.082
Acute Kidney Injury	41	1.3%	30	1.25%	4	1.59%	7	1.42%	0.876
Sepsis	293	9.3%	225	9.37%	26	10.32%	42	8.52%	0.712
Septic Shock	113	3.6%	86	3.58%	12	4.76%	15	3.04%	0.490
Superficial Incisional SSI	251	8.0%	192	8.00%	20	7.94%	39	7.91%	0.998
Wound Infection	23	0.7%	19	0.79%	1	0.40%	3	0.61%	0.737
Organ Space SSI	390	12.4%	292	12.16%	42	16.67%	56	11.36%	0.089
Dehiscence	39	1.2%	31	1.29%	4	1.59%	4	0.81%	0.595
Pulmonary Embolism	41	1.3%	32	1.33%	5	1.98%	4	0.81%	0.396
Deep Vein Thrombosis	167	5.3%	141	5.87%	9	3.57%	17	3.45%	0.040
Pancreatic Fistula	315	10.0%	245	10.25%	27	10.93%	43	9.01%	0.650

*SSI* surgical site infection.

**Fig. 2:**
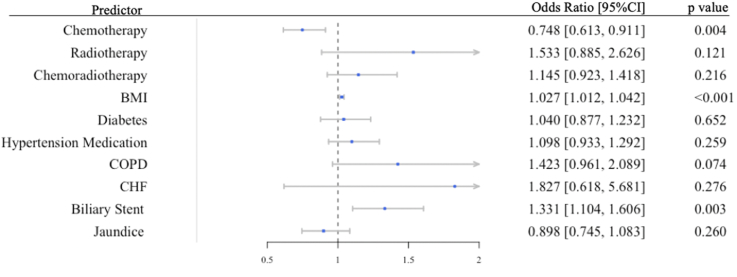
Preoperative Patient Characteristics as Predictors for 30-Day Major Complications. Generalized regression model was built to identify specific preoperative factors that predispose to early morbidities. Odds ratio were risk-adjusted by types of vascular resection. *BMI* Body Mass Index, *CI* Confidence Interval, *COPD* chronic obstructive pulmonary disease, *CHF* congestive heart failure.

**Fig. 3:**
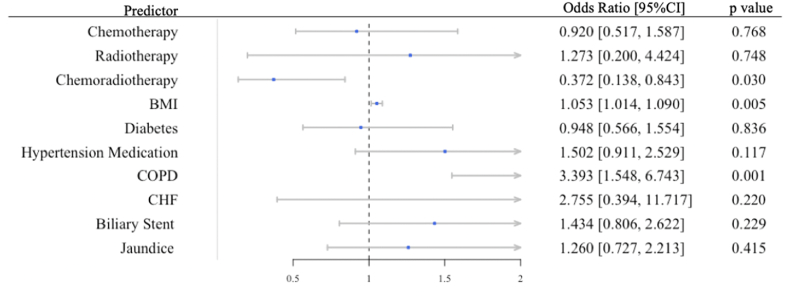
Preoperative Patient Characteristics and Diagnosis as Predictors for 30-Day Mortality. Generalized regression model was built to identify specific preoperative factors that predispose to early mortality. Odds ratio were risk-adjusted by types of vascular resection. *BMI* Body Mass Index, *CI* Confidence Interval, *COPD* chronic obstructive pulmonary disease, *CHF* congestive heart failure.

Risk factors for early major morbidity and mortality were identified through multivariable logistic regression models adjusted for types of vascular resections and comorbidities. Regardless of types of vascular resection, obesity, presence biliary stent preoperatively were independent positive risk factors that increased odds of developing any early major morbidity (Fig. 2). Neoadjuvant chemotherapy is a negative predictor for early morbidity. Obesity and COPD were predictors of 30-day mortality while neoadjuvant chemoradiotherapy were protective for early mortality (see Fig. 3).

## 4 Discussion

PDVR has been a subject of debate in the management of locally advanced and borderline resectable pancreatic tumors due to a poor prognosis. Previous multi-institutional studies have reported an increased incidence of complications and mortality in PDVR compared to pancreatoduodenectomy alone [14,15,19,20]. However, as resection with negative margin remains the only viable treatment with long-term survival benefits, PDVR has been increasingly performed for locally advanced pancreatic tumors. Previous studies have reported an increase of PDVR from 0.7% in 2000 to 6.0% in 2009 among patients requiring pancreatic resection for malignant pancreatic disease [14]. In the present study that included pancreatic resection cases for both benign and malignant pancreatic disease, there was also a significant increase in implementation of vascular resection. Despite an increase in cases with vascular resection, there does not appear to be any significant change in the rate of early morbidity and mortality over the years. Therefore, this study aims to investigate risk factors that predispose patients to early morbidity and mortality following PDVR.

Previous studies utilizing NSQIP data have disagreed on the significance of types of vascular resections in early morbidity or mortality rate. Zettervall reports a significantly higher early mortality rate with arterial procedures among pancreaticoduodenectomy from 2014 to 2015 [21]. Beane et al. reported no significant difference in overall morbidity and mortality rate between vein only resection and resection involving arterial structures following pancreaticoduodenectomy from 2011 to 2012 [22]. Both studies utilize a short study period with a smaller sample size. In the present study, we were able to separate vascular reconstructive procedures into vein only, artery only and concomitant vein and artery. Our results suggest that there was no significant difference in morality amongst different types of vascular resection in pancreaticoduodenectomy. Our finding suggests that anticipation of arterial resection should not be a deterring factor for patients requiring a more extensive pancreaticoduodenectomy.

Multivariable analyses suggest that a higher BMI might be a modifiable risk factor that contributes to early morbidity. However, obesity is not an independent predictor for early mortality. This is consistent with results of other studies that utilize datasets with well-balanced preoperative patient characteristics and pathological features demonstrating that obesity has no significant impact on short and long term survival [23]. Obesity is associated with fatty infiltration of the pancreas, which may lead to a greater risk of pancreatic fistula development [24].

Biliary stents have been proposed historically to provide symptomatic relief of jaundice and improve surgical outcomes by optimizing nutritional and metabolic functions [25]. However, there has been debate on the efficacy of preoperative biliary stent placement and associated postoperative septic complications [26,27]. Pancreatoduodenectomy patients who received preoperative biliary stent placement have approximately two to three times higher risk of developing wound infections compared to patients who did not undergo preoperative biliary decompression [26,28]. In the present multivariable model, preoperative biliary stent placement was identified as a risk factor of early postoperative morbidity but no impact on early mortality. These findings are consistent with a meta-analysis study that considered five randomized controlled studies suggesting that preoperative biliary stent placement should not be performed routinely given the complication rate and increased length of stay [29].

The present study showed higher incidence of deep vein thrombosis in PDVR involving vein reconstruction only (5.87%) compared to artery reconstruction only (3.57%) and concomitant vein and artery reconstruction (3.45%). Previous long term study has shown similar results with venous reconstruction resulting in higher rates of thrombosis events compared to arterial reconstruction [16]. The increased rate of thrombosis events in venous reconstruction can be secondary to the nature of low-flow system with relatively more extensive formation of collateral portal venous system [30]. Currently, there are no clear guidelines for anticoagulation following PDVR. Anticoagulation use in PDVR with venous resection and reconstruction have not shown significant benefit in decreasing thrombosis rates [31]. Furthermore, one systematic review indicated that patients who were on anticoagulation in the immediate post-operative period have significantly high incidence of bleeding with no significant benefit on prevention of portal vein thrombosis [32].

The value of neoadjuvant therapies in resectable and borderline resectable pancreatic cancer has been a topic of debate. In the present study, we categorized neoadjuvant therapies into chemotherapy, radiotherapy, and chemoradiotherapy. After adjusted for types of vascular resections and comorbidities, patients who received chemotherapy were found to have lower risks of developing early morbidities while patients who received chemoradiotherapy have lower risks of 30-day mortality. Earlier studies have raised concern for increased complication rates and minimal survival benefit in patients who received neoadjuvant therapies [[7], [8], [9]]. Therefore, neoadjuvant therapies were only recommended in higher-volume hospitals especially in the setting of borderline resectable pancreatic tumor [6]. However, recent studies have shown significantly increased survival benefits in neoadjuvant therapies. A systematic review that included 38 studies demonstrated significant improvement in median survival in patients receiving neoadjuvant therapies followed by resection versus patients receiving immediate surgery (26.1 months versus 15.0 months). There was also a significantly higher R0 rate in the neoadjuvant groups [10]. Furthermore, a prospective clinical trial (PREOPANC-1) that randomized patients into preoperative chemoradiotherapy and immediate surgery reported an overall significant survival benefits in patients who received neoadjuvant therapies (median 17.1 vs. 13.5 months) as well as an increase in R0 resection rate (65% vs. 31%) [11]. Because of the emerging new evidence of the potential benefit of neoadjuvant therapies, recent recommendations of PDVR suggest an additional rationale for neoadjuvant treatment in borderline resectable pancreatic cancer [6,12].

There are limitations in analyzing effect of neoadjuvant therapies on postoperative outcome in current study. As this study utilized a multi-intuitional dataset, the neoadjuvant therapy protocols included may have been heterogenous and specific protocols used for each patient were not available for analyses. Nevertheless, despite the acknowledgement that most studies to date report heterogenous neoadjuvant protocols, current NCCN guidelines recommend neoadjuvant therapy instead of immediate resection to improve R0 resection rates [6].

Other limitations in the present study include inability to evaluate long-term survival and outcomes. Information for vascular procedures were limited to involvement of vein, artery or vein and artery; details of portal vein resection grades or extent of vascular procedure could not be analyzed. Information regarding reconstruction methods such as primary repair or autologous venous patch were also unavailable. While the procedure targeted dataset provided some pancreatectomy specific complications such as pancreatic fistula, other known complications such as portal vein thrombosis were not recorded. Furthermore, whether the vascular resection was planned preoperatively or an intra-operative decision due to events in the operating room were unknown.

While the results of this study should be interpreted within the context of the above limitations, this study presents valuable findings with the largest and most up to date cohort of patients undergoing PDVR. This study identifies specific preoperative patient-specific factors and modifiable risk factors, such as higher BMI and preoperative biliary stent placement as risk factors and neoadjuvant therapy as a negative predictor. The goal is to create a patient-centered and personalized perioperative planning to optimize surgical outcomes.

## 5 Conclusion

This study reports the largest multi-institutional series that investigates the outcomes following PDVR. The results suggest that, despite a trend of increase utilization of PDVR in patients undergoing pancreatic resection, the incidence of 30-day morbidity and mortality remains unchanged. The study identified preoperative risk factors that predispose patients to early morbidity and mortality. Chemotherapy was an independent predictor for decreased early morbidity, whereas chemoradiotherapy was an independent predictor for improved early survival. The results of this study may assist decision making for perioperative management to improve overall survival following PDVR and guide areas of focus for future studies.

## Provenance and peer review

Not commissioned, externally peer-reviewed.

## Ethical approval

Institutional Review Board (IRB) of the Icahn School of Medicine at Mount Sinai (IRB-20-02957).

## Sources of funding

None.

## Author contribution

Study design: I. Michael Leitman, TiffanyY. Lim.

Data Collection: I. Michael Leitman.

Data analysis: Tiffany Y. Lim.

Writing: I. Michael Leitman, Tiffany Y. Lim.

## Registration of research studies

1. Name of the registry: Research Registry.

2. Unique Identifying number or registration ID: researchregistry6923.

3. Hyperlink to your specific registration (must be publicly accessible and will be checked):

https://www.researchregistry.com/browse-the-registry#home/registrationdetails/60d5ef54fe99b3001ee01db2/

## Guarantor

I. Michael Leitman, MD.

## Consent

N/A.

## Declaration of competing interest

None.
